# Destruction of Forests

**Published:** 1883-12

**Authors:** 


### DESTRUCTION OF FORESTS.

SEVERAL years ago there was great lamentation over the destruction of the buffalo, until some political economist suggested that an ox required no more food than a buffalo, and that the country would be richer by hundreds of millions of dollars if the buffalo could be exterminated and their places occupied by good breeds of cattle. We remarked to a friend, recently, that the supply of good pine lumber was nearly exhausted in this country. He said he was glad to hear that was the case, for so long as the pine lumber lasted it would exclude from the market various materials that have been experimented with sufficiently to prove that they are in all respects equal to, and on many accounts superior to lumber.

It seems more than probable that ten years after the pine lumber shall have disappeared there will be but few regrets for its loss; and that in the next century a new growth will have taken the place of the old, undisturbed by the axe of the pioneer. The history of the lumber trade may come to resemble in that respect the history of the whale oil trade. Petroleum destroyed that business, as straw-lumber, paper maché and similar manufactures are likely to destroy the lumber trade.

There can be no doubt but that the wholesale destruction of the forests in this country is doing great injury to its agricultural interests, and that it should be stopped, and particularly in the older States, where hardly any trees are left to make new forests, and where the soil is so thin that the land becomes a desert when unprotected by trees. But in unsettled portions of the country to which immigration is not attracted it is probable that the evil will work out its own cure through natural causes.

BREATHE not a sentiment, say not a word, give not an expression of the countenance that will offend another, or send a thrill of pain through his bosom. We are surrounded by sensitive hearts, which a word, or look even, might fill to the brim with sorrow.

